# Childhood unpredictability and sleep quality in adulthood: the mediating roles of life history strategy and perceived stress

**DOI:** 10.3389/fpsyg.2024.1347365

**Published:** 2024-04-18

**Authors:** Wei Qi, Xiangyang He, Zhechen Wang

**Affiliations:** ^1^Department of Psychology, Zhejiang Sci-Tech University, Hangzhou, Zhejiang, China; ^2^School of Social Development and Public Policy, Fudan University, Shanghai, China; ^3^School of Psychology, The University of Queensland, Brisbane, QLD, Australia

**Keywords:** early environmental risk, childhood unpredictability, sleep quality, life history strategy, perceived stress

## Abstract

**Background:**

Early environmental risk have been found to be related to lifelong health. However, the impact of childhood unpredictability, a type of early environmental risk, on health, especially on sleep quality in adulthood, has not been adequately studied. The present study aimed to examine the relationship between childhood unpredictability and sleep quality in adulthood and to explore the possible mediating roles of life history strategy and perceived stress.

**Methods:**

A cross-sectional study was conducted on 472 participants from a university in Zhejiang Province, China. The questionnaire inquired about demography, childhood unpredictability, life history strategy (Mini-K), perceived stress (14-item Perceived Stress Scale), and Sleep Quality (Pittsburgh Global Sleep Quality Index).

**Results:**

Higher childhood unpredictability was significantly associated with worse sleep quality in adulthood. Moreover, the link between higher childhood unpredictability and worse sleep quality in adulthood was explained by the chain mediation of life history strategy and perceived stress.

**Conclusion:**

In line with the life history theory, individuals who have experienced higher unpredictability in childhood tend to develop a faster life history strategy and become more sensitive to stress in adulthood, and subsequently suffer a decrease in sleep quality.

## 1 Introduction

Early life environment exerts a critical impact on health across the lifespan. Early environmental stressors, such as childhood adversity, low childhood socioeconomic status, and high childhood unpredictability, have been shown to have negative effects on overall health (Galobardes et al., 2004; Adler and Stewart, 2010; Schulz et al., 2012; Doom et al., 2017; Maner et al., 2022). Individuals who have experienced greater adversity (such as emotional neglect and abuse) or have faced more physical and psychological stress in childhood are less likely to engage in health behaviors and have a higher risk of cardiovascular and other diseases in adulthood (Doom et al., 2017). In addition, other literature reports that lower childhood socioeconomic status led to worse health and a shorter life expectancy (Galobardes et al., 2004; Adler and Stewart, 2010; Schulz et al., 2012). Overall, prolonged exposure to predictably harsh environments early in life can have a negative impact on lifelong health.

Recent studies have found that childhood unpredictability, an important but often neglected part of early life environmental risk, also has a critical impact on an individual’s health throughout the lifespan (Doom et al., 2015; Maner et al., 2022). Childhood unpredictability, which is distinct from childhood adversity and childhood socioeconomic status, represents the stochastic variability of environmental conditions, that is, uncertainty and instability in time or space due to early harshness (Ellis et al., 2009) including frequent changes in ecological and familial conditions (e.g., residential changes, resource fluctuations, parental inconsistency; Mittal et al., 2015; Luo et al., 2020). Maner et al. (2022) found that perceiving the environment to be more uncertain, unstable, or uncontrollable in childhood is related to worse health in adulthood. Doom et al. (2015) noted that early (between the ages of 0 and 5) exposure to greater unpredictability predicted more externalizing behaviors as well as greater alcohol and marijuana use at age 16. Similarly, family environments with higher levels of conflict and lower levels of warmth and affection led to more disruptions in psychosocial functioning (especially emotional processing and socialization), stress-responsive bioregulatory systems (including sympathetic-adrenomedullary and hypothalamic–pituitary-adrenocortical functioning), and more unhealthy behaviors such as substance abuse, thereby impairing health in adulthood (Repetti et al., 2002; Taylor et al., 2004; Loucks et al., 2011).

The decline in sleep quality in adulthood is one of the significant consequences of stressors in early life environment (Koskenvuo et al., 2010; Chapman et al., 2011; Kajeepeta et al., 2015; Wang et al., 2016). A large number of studies have examined the effects of childhood adversity and childhood socioeconomic status on sleep quality, with largely consistent results (Koskenvuo et al., 2010; Tomfohr et al., 2010; Chapman et al., 2011; Greenfield et al., 2011; Ramsawh et al., 2011; Baiden et al., 2015; Kajeepeta et al., 2015; Wang et al., 2016; Brindle et al., 2018; Counts et al., 2018; John-Henderson, 2020). However, much fewer studies have examined the effect of childhood unpredictability on sleep quality. A recent study in the Israeli Jewish community examined the effects of multiple early environmental stressors on people’s sleep quality during the COVID-19 epidemic (Haimov et al., 2022). Results showed that higher childhood unpredictability, as a separate and independent factor, was associated with a decrease in sleep quality and an increase in insomnia symptoms, and the association was completely mediated through COVID-19-related anxiety, suggesting that childhood unpredictability could contribute to a decrease in sleep quality during stressful periods. However, this study did not explain why higher early environmental stress led to higher COVID-19-related anxiety. In other words, it was still not clear why people who have experienced higher unpredictability in childhood were more able to perceive stress in the current environment and were more likely to be affected by this perceived stress. In our study, we attempted to answer this question from a life history perspective. Specifically, we argued that perceiving a greater stress might be an adaptive manifestation of a fast life history strategy (Mittal et al., 2015). It could enhance outward/external vigilance (Proffitt Leyva and Hill, 2018; Cabeza de Baca and Albert, 2019), but might also cause sleep disturbance.

Moreover, it is worth noting that the measure of childhood unpredictability in previous studies has limitations. For instance, in the study of Haimov et al. (2022), childhood unpredictability was assessed by changes in resource availability or residential environment as well as changes inside the family (i.e., an increase or decrease in the number of family members). This measurement did not account for inconsistencies in parenting behavior (i.e., parental emotional and behavioral unpredictability), which was also one part of the causal factors for unpredictability (Ross and Hill, 2000, 2002). In this regard, our current study employed a more multidimensional measure of unpredictability.

In the following, we will first introduce the key points of life history theory and then argue for possible pathways by which childhood unpredictability may influence sleep quality in adulthood.

### 1.1 Life history theory

Derived from the evolutionary psychology perspective, life history theory (LHT) provides a framework for describing how individuals invest their limited resources (energy, time, etc.) into different areas that are closely related to their life functions: maintenance and reproduction (Chisholm, 1999; Rowe, 2000; Kaplan and Gangestad, 2015). To maximize reproductive success, individuals make adaptive trade-offs between maintenance and reproduction based on environmental cues which consist of environmental harshness and unpredictability (Griskevicius et al., 2011a,b, 2013; Ellis et al., 2009). This tendency to trade-off is conceptualized as a life history strategy - a continuous spectrum with “fast life history strategy” and “slow life history strategy” as its two poles. For example, in a harsh and unpredictable environment, death may occur before long-term investments in growth and maintenance are rewarded, thus, it is more adaptive to prioritize rapid maturation and reproduction over maintenance, and individuals thus are likely to develop a fast life history strategy which characterized by a focus on immediate benefits and risk-seeking. In contrast, individuals who grow up in a relatively safe, stable, and predictable environment are more likely to focus on physical nurturing and skill development and invest more resources in growth and maintenance, thereby developing a slow life history strategy (Ellis, 2004; Belsky, 2010; Griskevicius et al., 2011a,b, 2013).

The function of “maintenance” is achieved through diverting resources to critical systems or through recovery and regeneration mechanisms, which necessarily reduces investment in current reproduction (Ellis et al., 2009). Particularly, sleep is an investment in maintenance through mechanisms dedicated to recovery and regeneration (Cribbet et al., 2014; Cao et al., 2020). Therefore, to some extent, differences in sleep patterns and sleep quality could reflect differences in individuals’ choices of life history strategy (Dishakjian et al., 2020). For instance, Dishakjian et al. (2020) noted that a slow life history strategy is conducive to sleep quality, whereas a fast life history strategy is associated with worse sleep quality such as delayed sleep onset latency, higher sleep–wake instability, and more wakefulness and variability after sleep.

In short, sleep quality is likely to be affected by life history strategy. Notably, as mentioned earlier, childhood environmental stressors (such as unpredictability) can affect sleep quality as well. Also, childhood unpredictability is an antecedent variable that shapes life history strategy. Therefore, we propose that the link between childhood unpredictability and sleep quality may be explained by life history strategy.

### 1.2 Mechanisms by which childhood unpredictability affects sleep

Though prior work has suggested the possibility that sleep quality might be affected by life history strategy, the underlying mechanism is still not clear. To address this issue, we propose that individuals with a faster life history strategy should have higher sensitivity to current environmental stressors, which will in turn affect sleep quality in adulthood.

In line with the life history strategy, individuals’ sensitivity to environmental stressors plays an important role in evolutionary adaptations. Namely, humans have often faced many exogenous stressors during their evolutionary history, such as food shortages, natural disasters, and violent conflicts (Lin and Wang, 2015). Perceiving these stressors sensitively is beneficial for coping with them more quickly, thereby enhancing human survival and successful reproduction, reflecting evolutionary adaptations (Mittal et al., 2015). Notably, sleep patterns are also a manifestation of the evolutionary adaptations described above. In particular, shorter sleep durations and higher proportions of rapid eye movement sleep may be related to advantages for better coping with environmental stressors derived from learning, cooperation, and defense against attacks (Samson and Nunn, 2015; Nunn et al., 2016). Moreover, harshness and unpredictability in early life promote the formation of a faster life history strategy. Individuals with a faster life history strategy are more sensitive to stress cues in their environments because they have experienced more environmental risk and have adapted to the idea that future environments will also be harsh and unpredictable, which can help them better prepare for challenges ahead (Figueredo et al., 2004; Belsky, 2010; Lin and Wang, 2015; Birkás et al., 2018; van der Linden et al., 2018). The sensitivity to environmental risk and stressors, on the one hand, brings the benefit of “adaptive calibration” (Ellis and Del Giudice, 2013). On the other hand, however, it also comes at the expense of sleep duration and sleep quality (Taylor et al., 2004; Sanford and Wellman, 2012; John-Henderson et al., 2018; Haimov et al., 2022). For instance, Sanford and Wellman (2012) found that experiencing uncontrollable stress and warnings of stress significantly reduced the length of sleep, especially that of rapid eye movement sleep. John-Henderson et al. (2018) found that for college students, psychological distress experienced during the stressful transition (early in college life) mediated the relationship between childhood adversity (primarily emotional neglect) and changes in adulthood sleep quality. Therefore, it is reasonable to assume that individuals’ perception of stress might be underlying the link between life history strategy (which is shaped by childhood unpredictability) and sleep quality in adulthood.

### 1.3 The current study

The current study aims to examine the relationship between childhood unpredictability and sleep quality in adulthood. In line with the life history theory, we will further explore the mechanisms by which childhood unpredictability influences sleep quality in adulthood. In particular, we expect that individuals who experience higher unpredictability in early life tend to develop a faster life history strategy and individuals with a faster life history strategy should be more sensitive to stress later in life which, in turn, will undermine their sleep quality in adulthood. Together, we propose the following hypotheses:

*H1*: Higher childhood unpredictability is associated with worse sleep quality in adulthood.

*H2*: The relationship between higher childhood unpredictability and worse sleep quality in adulthood is explained by the chain mediation of faster life history strategy and higher perceived stress.

## 2 Methods

### 2.1 Participants

This study was approved by the Research Ethics Committee of the first author’s University. According to the calculations of the Monte Carlo asymptotic method, at least 195 subjects were required in this study to achieve the maximum statistical test power in this chain mediation model (Weaver and Wuensch, 2013; Schoemann et al., 2017; Sim et al., 2021). All data were collected through a web-based survey platform (Questionnaire Star^1^) in China. All participants voluntarily and anonymously participated in this study and signed an informed consent form on the Internet. A total of 602 questionnaires were collected. After attention check and data cleaning (Goodman et al., 2012), the final sample consisted of 472 valid questionnaires, including 252 females and 220 males.

### 2.2 Measures

#### 2.2.1 Sleep quality

The Pittsburgh Global Sleep Quality Index (PSQI) was used as a measure of participants’ overall sleep quality (Buysse et al., 1989). The scale consisted of 19 items and was used to derive a total of seven component scores: sleep quality, sleep latency, sleep duration, habitual sleep efficiency, sleep disturbances, sleep medications, and daytime dysfunction. The scores of the seven components are combined to produce an overall PSQI score. The range of possible values is from 0 to 21. The higher the score, the worse the sleep quality. In the current study, the Cronbach’s alpha was 0.69 and the Spearman-Brown coefficient was 0.65.

#### 2.2.2 Childhood unpredictability

According to previous research (Luo et al., 2020), the unpredictability of one’s early experience contained two dimensions: the unpredictability of the residential environment (e.g., people often moved in and out of my house on a pretty random basis; Mittal et al., 2015) and the unpredictability of parents’ emotions and behavior (e.g., Whether or not my parents disciplined me when I acted up depended on their mood at the time; Ross and Hill, 2000). Therefore, we used the revised environmental unpredictability questionnaire (Luo et al., 2020) to measure participants’ childhood unpredictability. This questionnaire included three items measuring residential unpredictability and six items measuring parental emotional and behavioral unpredictability. All items were scored on a 5-point Likert scale (1 = strongly disagree, 5 = strongly agree). In our study, the two dimensions were highly correlated (*r* = 0.59, *p* < 0.05) and were combined to obtain an overall score of environmental unpredictability, with higher scores representing higher unpredictability of the childhood environment. For the whole questionnaire, the Cronbach’s alpha was 0.89 and the Spearman-Brown coefficient was 0.77.

#### 2.2.3 Life history strategy

The Mini-K version of the Arizona Life History Battery was used to assess participants’ choice of life history strategy (Figueredo et al., 2014). The scale consisted of 20 questions including six dimensions: insight, planning, and control; parental relationship quality; friend social contact/support; family social contact/support; pair-bonding; and community involvement, using a 7-point Likert scale (1 = strongly disagree, 7 = strongly agree). The higher the mini-k score, the slower the life history strategy. In the current study, the Cronbach’s alpha was 0.89 and the Spearman-Brown coefficient was 0.82.

#### 2.2.4 Perceived stress

The 14-item Perceived Stress Scale (PSS) was used to assess the extent to which life situations were perceived as stressful (Cohen et al., 1983). The scale was a 5-point Likert scale (1 = strongly disagree, 5 = strongly agree). The higher the score, the greater the stress participants perceived in their life situations. In the current study, the Cronbach’s alpha was 0.70 and the Spearman-Brown coefficient was 0.77.

### 2.3 Data analysis

SPSS 26.0 was used for all data cleaning, common method bias, descriptive statistical analyses, and correlation analysis in this study. Based on the results of the correlation analysis and the hypotheses presented above, we developed a linear regression model to test the relationship between childhood unpredictability and sleep quality in adulthood. The chain mediation path was validated using the PROCESS Macro for SPSS (Hayes, 2013). Model 6 was specified because it accounted for the order of mediator variables. Namely, it assumed a causal relationship (rather than a parallel relationship) between the first and the second mediator variables. The mediation bootstrapping analysis was conducted using 5,000 resamples and a 95% bias-corrected confidence interval (CI). Age and gender were considered as covariates in the regression model, gender was treated as a dummy variable in mediation analysis (Female = 0, Male = 1). Besides, considering the effect of current socioeconomic status on sleep quality, as in the previous study (e.g., Moore et al., 2002; Marco et al., 2012), we also used family income monthly to represent current socioeconomic status and added it as a covariate in the current study.

## 3 Results

### 3.1 Common method bias test

Harman’s (1967) one-way test was selected to evaluate the likelihood of common method bias in this study, and four self-reported variables in the study were analyzed together using exploratory factor analysis. The results showed that the percentage of variance explained by the first component was 19.1%, which was less than 30%. Therefore, it can be concluded that there was no serious common method bias in this study.

### 3.2 Descriptive statistics and correlation analysis

The results of descriptive statistics were listed in Table 1, and partial correlations between main variables were reported in Table 2.

**Table 1:** Descriptive statistics.

	*n*	M(SD)	%(*n*)	Range
Age	472	21(1.6)		18–28
Gender (%female)	472		46.6(252)	
Family income	472			
0–2,500			1.5(7)	
2,501–7,500			23.7(112)	
7,501–12,500			34.1(161)	
12,501–17,500			22.5(106)	
17,501–22,500			10.0(47)	
>22,500			8.3(39)	
PSQI	472	4.59(2.71)		0–14
Childhood unpredictability	472	24.62(7.00)		9–45
Mini-K	472	104.13(15.87)		30–140
Perceived stress	472	41.31(8.54)		17–69

PSQI, Pittsburgh sleep quality index; Family income was measured by family income monthly (1 RMB as a unit).

**Table 2:** Partial correlations.

	1	2	3	4
1. PSQI	-			
2. Childhood unpredictability	0.24^***^	-		
3. Mini-K	−0.25^***^	−0.23^***^	-	
4. Perceived stress	0.40^***^	0.38^***^	−0.60^***^	-

*p < 0.05, **p < 0.01, ***p < 0.001. Age, gender, and family income were the control variables.

As shown in Table 2, there were significant correlations between the main variables. Specifically, higher childhood unpredictability was significantly correlated with higher scores of PSQI, therefore supporting our hypothesis 1 that higher childhood unpredictability is associated with worse sleep quality in adulthood. Higher childhood unpredictability was also significantly correlated with greater perceived stress and a slower life history strategy (as indicated by higher mini-K). The correlation between Mini-K and PSQI was negative, suggesting that a slower life history strategy was associated with better sleep quality. The correlation between perceived stress and PSQI was positive, suggesting that greater perceived stress was associated with worse sleep quality.

### 3.3 Analysis of chain-mediated effects

Based on the results of correlation analyses, a chain mediation model was developed in this study to test the mediating effect. In this model, sleep quality in adulthood was the outcome variable, childhood unpredictability was a predictor variable, and life history strategy and perceived stress were mediating variables, age, gender, and family income were included as covariates. The results showed that the model was valid [*R*^2^ = 0.19, *F*(6, 465) = 17.93, *p* < 0.001], and the model explained 19% of the variance in PSQI scores, which is 16% more of the variance than the covariate-only model [*R*^2^ = 0.03, *F*(3, 468) = 4.05, *p* < 0.01].

**Figure 1:**
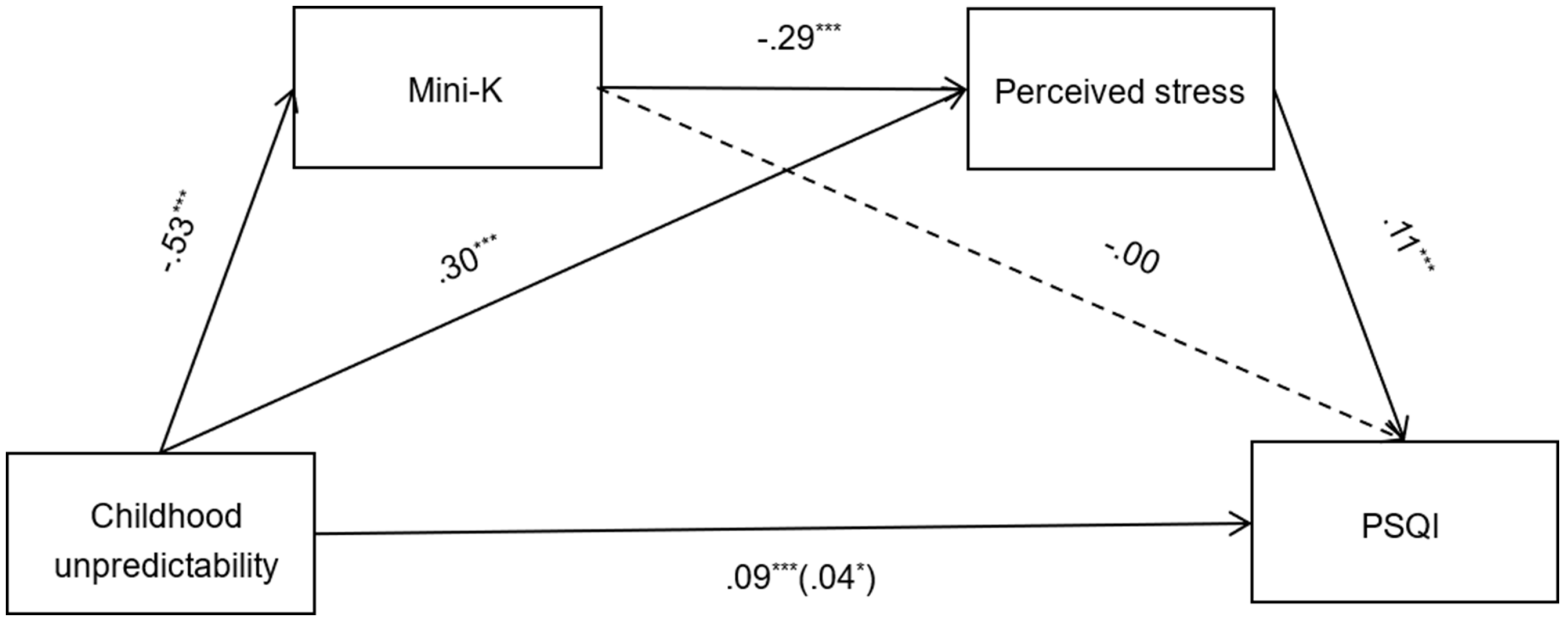
Chain mediation model with unstandardized coefficient. *p < 0.05, **p < 0.01, ***p < 0.001. Age, gender, and family income were included as covariates in the model, which were not drawn in the figure. On the lower path, the total effect was shown outside the parentheses, the direct effect was shown inside the parentheses.

The direct effects and indirect effects were shown in Figure 1. Results showed that the total effect (*B* = 0.09, 95%CI = [0.06, 0.13]) and direct effect (*B* = 0.04, 95%CI = [0.01, 0.08]) of childhood unpredictability on PSQI in adulthood was statistically significant, which meant that higher childhood unpredictability directly predicted worse sleep quality (as indicated by higher PSQI). Notably, the indirect pathway from childhood unpredictability to Mini-K to perceived stress to PSQI was significant (*B* = 0.02, 95%CI = [0.01, 0.03]). Therefore, our hypothesis 2 was supported: higher childhood unpredictability indirectly predicted worse sleep quality in adulthood via faster life history strategy and higher perceived stress.

In addition, the indirect effect of the pathway from childhood unpredictability to perceived stress to PSQI was significant (*B* = 0.03, 95%CI = [0.02, 0.05]), suggesting that higher childhood unpredictability could also indirectly predict worse sleep quality in adulthood through higher perceived stress. However, the indirect effect of the path from childhood unpredictability to Mini-K to PSQI was not significant (*B* = 0.00, 95%CI = [−0.01, 0.01]), that was, higher childhood unpredictability could not indirectly predict worse sleep quality in adulthood only through faster life history strategy.

## 4 Discussion

A large number of previous studies have examined the association between early life environment and future overall health (Galobardes et al., 2004; Adler and Stewart, 2010; Benham, 2010; Chapman et al., 2011; Greenfield et al., 2011; Schulz et al., 2012; Doom et al., 2017; Maner et al., 2022). In line with the life history theory, the present study explored the relationships between childhood unpredictability, adaptive efforts (life history strategies and perceived stress), and adverse health outcomes (especially sleep health).

First, our results showed that there was a significant correlation between early unpredictability and sleep quality in adulthood, therefore supporting our hypothesis 1. Given previous studies have mostly focused on the influence of childhood socioeconomic status and childhood adverse on future sleep quality (Galobardes et al., 2004; Koskenvuo et al., 2010; Tomfohr et al., 2010; Chapman et al., 2011; El Shakankiry, 2011; Ramsawh et al., 2011; Baiden et al., 2015; Kajeepeta et al., 2015; Wang et al., 2016; Counts et al., 2018; John-Henderson, 2020), our study revealed that childhood unpredictability is also a significant risk factor for predicting sleep disturbance in adulthood. This result suggested that, besides harsh environments, dynamic changes in the family environment could also exert profound impacts on individual health. Notably, there is nuance in our findings and previous findings as well. Specifically, in Haimov et al.’s (2022) regression model, childhood unpredictability did not directly affect sleep quality in adulthood, but was completely mediated through levels of anxiety during the COVID-19 crisis. In contrast, the results of our present study supported a direct effect of childhood unpredictability on adult sleep quality. This may be due to the fact that the present study also included the unpredictability of parenting emotions and behaviors when measuring childhood unpredictability. The unpredictability of parental emotions and behaviors, in perspective of allostatic load (McEwen and Stellar, 1993; Guidi et al., 2020), maybe a cumulative stressor causing wear and tear on physiological systems (neuroendocrine, immune, etc.), and their negative impact on sleep quality is more immediate and does not need to be mediated by adaptive strategies or the environment in adulthood.

Second, our results showed that childhood unpredictability influenced sleep quality in adulthood via life history strategy and stress perception, therefore supporting our hypothesis 2. To some extent, this was consistent with previous studies suggesting that childhood unpredictability was a crucial antecedent variable for life history strategies (Ellis et al., 2009; Griskevicius et al., 2011b; Kaplan and Gangestad, 2015; Luo et al., 2020). Similarly, our finding was also consistent with prior work showing that a fast life history led to increased perceived stress (Jonason et al., 2016; Birkás et al., 2018) and increased stress perception caused a decrease in sleep quality (Sanford and Wellman, 2012; John-Henderson et al., 2018). Taking one step further, our study has integrated these processes. In particular, our results showed that early unpredictability affects sleep quality in adulthood via the chain mediation of life history strategy and stress perception. These results suggested that, from the perspective of life history theory, people exposed to unpredictable environments in early life may develop stress-adapted strategies to gain advantages for future harsh and unpredictable environments (Young et al., 2020). One of such advantages is the alertness and sensitivity to the current environment, especially to stressors, which helps individuals to quickly recognize and respond to possible hazards. Moreover, although developing adaptations to high-stress environments would allow individuals to “make the most of a bad situation” (i.e., mitigate unavoidable and fatal physical costs), such adaptations may come at health costs. Specifically, individuals’ hypersensitivity to environmental stressors may disturb their sleep quality. For instance, perceived stress in the current environment is likely to cause an increase in the individual’s anxiety level, which can reduce sleep duration in order to think about or implement coping strategies. Also, perceived stress may trigger a number of physiological responses that reduce sleep depth in order to make the organism more alert. In this sense, our findings provided an explanation for Haimov et al.’s (2022) findings that childhood unpredictability led to reduced sleep quality during stressful periods, which has the effect of increasing an individual’s advantage in adapting to the current environment, in which life-history strategies play a role of adaptive calibration.

In addition, there may be other indirect pathways in how early life unpredictability affects sleep quality in adulthood as well. Namely, besides the chain mediation of life history strategy and perceived stress, the mediation effect of perceived stress alone underlying the relationship between higher childhood unpredictability and worse sleep quality in adulthood was also significant. This finding suggests that early stressors may alter individuals’ susceptibility to stress through other pathways (besides through life history strategy). For instance, repeatedly experiencing and dealing with stressful events may increase the accessibility of an individual’s negative schema (focusing on negative aspects in oneself and the world, ignoring positive aspects), resulting in a stress-sensitization effect (Hammen et al., 2000).

Overall, our study has both theoretical and practical implications. First, it extends previous literature on early environmental risk and health in adulthood. Specifically, in line with the life history theory, our model provides an explanation of how childhood unpredictability affects sleep quality in adulthood. Second, traditional views of sleep medicine have primarily used a pathophysiological approach to understanding and treating sleep disorders. However, we caution that growing up in families with variable discipline and inconsistent parenting styles may play a role in affecting sleep quality as well. In this regard, the importance of maintaining a stable and consistent parenting environment, or increasing resilience, should be emphasized in home or school education in order to improve the sleep quality of the population.

Admittedly, our study has some limitations. First, since our study design is cross-sectional, it is unable for us to infer the causality between variables. Future research could adopt a longitudinal design or use experimental studies to determine the causal relationship. Second, in our study, we used college student participants’ retrospective self-reported childhood unpredictability as the predictor variable. However, the accuracy of individuals’ memories of their early life circumstances may be negatively affected by time, and this effect may be particularly strong for older participants (Counts et al., 2018). Therefore, future research should employ objective indicators of childhood unpredictability and examine our findings in a broader population. In a related vein, in our study, participants’ sleep quality was measured using the PSQI, which largely relied on self-reports as well. Therefore, it might be beneficial for future research to include more objective sleep measures such as actigraphy and so on. Third, since our study was conducted during the COVID-19 pandemic, participants might experience unpredictability in terms of the outbreak of the pandemic as well as the changing pandemic prevention and control policies in China (Yang et al., 2021; Wang and Yin, 2023). This unpredictability at present might also impact individuals’ sleep quality. Thus, it would be important for future research to separate the potential influence of present unpredictability from childhood unpredictability as well (e.g., to replicate the study when the pandemic ends).

## 5 Conclusion

Based upon an evolutionary psychology perspective, our present research underscores the importance of considering childhood experiences when diagnosing physiological and psychological symptoms in adulthood. In particular, our findings shed light on a potential mechanism: childhood unpredictability shapes individuals’ life history strategies, subsequently impacting their perceived stress and ultimately influencing their sleep quality. Taken together, our study suggests the prospect of improving sleep quality by intervening at various points within this mechanism.

## Data availability statement

The datasets presented in this study can be found in online repositories. The names of the repository/repositories and accession number(s) can be found in the article/Supplementary material.

## Ethics statement

The studies involving human participants were reviewed and approved by the Ethics Committee for Psychological Research at the Zhejiang Sci-Tech University (Approval number: 202310H002). The participants provided their written informed consent to participate in this study.

## Author contributions

WQ: Conceptualization, Data curation, Formal analysis, Funding acquisition, Investigation, Methodology, Project administration, Resources, Software, Validation, Visualization, Writing – original draft. XH: Data curation, Investigation, Writing – original draft. ZW: Supervision, Conceptualization, Funding acquisition, Validation, Writing – review & editing.

## Footnotes

^1^ http://www.wjx.cn
