# Supplementary material for: Childhood unpredictability and sleep quality in adulthood: the mediating roles of life history strategy and perceived stress
Source: Front Psychol. 2024 Apr 18;15:1347365. doi: 10.3389/fpsyg.2024.1347365 (PMC11063338; doi:10.3389/fpsyg.2024.1347365)
Supplement: Supplementary file 1 [file Data_Sheet_1.zip › measures_Chinese-English_revised.docx]

**Demographic information**

1. 您的年龄是？

1、What is your age? Please write down the number ____

**2、您的性别是？**

1. What is your gender?
2. 男

Male

1. 女

Female

**3、您当前的家庭月收入为？**

4、What is your current monthly household income?

1. 0-2500元

0-2500¥

1. 2501-7500元

2501-7500¥

1. 7501-12500元

7501-12500¥

1. 12501-17500元

12501-17500¥

1. 17501-22500元

17501-22500¥

1. 22500元以上

>22500¥

**The Pittsburgh Global Sleep Quality Index (PSQI; Buysse et al., 1989)**

1. **下面一些问题是关于您最近1个月的睡眠情况，请选择或填写最符合您近1个月实际情况的答案。**

The following questions relate to your usual sleep habits during the past month only. Your answers should indicate the most accurate reply for the *majority* of days and nights in the past month. Please answer all questions.

1. 近1个月，晚上上床睡觉通常 ____点钟。
2. During the past month, when have you usually gone to bed at night? Usual bed time ___
3. 近1个月，从上床到入睡通常需要____分钟。
4. During the past month, how long (in minutes) has it usually take you to fall asleep each night? Number of minutes___
5. 近1个月，通常早上____点起床
6. During the past month, when have you usually gotten up in the morning? Usual getting up time__
7. 近1个月，每夜通常实际睡眠_____小时(不等于卧床时间)。
8. During the past month, how many hours of actual sleep did you get at night? (This may be different than the number of hours you spend in bed.) Hours of sleep per night__

对下列问题请选择1个最适合您的答案。

For each of the remaining questions, check the one best response. Please answer all questions.

1. 近1个月 ，因下列情况影响睡眠而烦恼:
2. During the past month, how often have you had trouble sleeping because you...
3. 入睡困难(30分钟内不能入睡)⑴无 ⑵〈1次/周 ⑶1-2次/周 ⑷≥ 3次/周

Cannot get to sleep within 30 minutes (1) Not during the past month (2) Less than once a week (3) Once or twice a week (4) Three or more times a week

1. 夜间易醒或早醒 ⑴无 ⑵〈1次/周 ⑶1-2次/周 ⑷≥ 3次/周

Wake up in the middle of the night or early morning (1) Not during the past month (2) Less than once a week (3) Once or twice a week (4) Three or more times a week

1. 夜间去厕所 ⑴无 ⑵〈1次/周 ⑶1-2次/周 ⑷≥ 3次/周

Have to get up to use the bathroom (1) Not during the past month (2) Less than once a week (3) Once or twice a week (4) Three or more times a week

1. 呼吸不畅 ⑴无 ⑵〈1次/周 ⑶1-2次/周 ⑷≥ 3次/周

Cannot breathe comfortably (1) Not during the past month (2) Less than once a week (3) Once or twice a week (4) Three or more times a week

1. 咳嗽或鼾声高 ⑴无 ⑵〈1次/周 ⑶1-2次/周 ⑷≥3次/周

Cough or snore loudly (1) Not during the past month (2) Less than once a week (3) Once or twice a week (4) Three or more times a week

1. 感觉冷 ⑴无 ⑵〈1次/周 ⑶1-2次/周 ⑷≥3次/周

Feel too cold (1) Not during the past month (2) Less than once a week (3) Once or twice a week (4) Three or more times a week

1. 感觉热 ⑴无 ⑵〈1次/周 ⑶1-2次/周 ⑷≥3次/周

Feel too hot (1)Not during the past month (2) Less than once a week (3) Once or twice a week (4) Three or more times a week

1. 做恶梦 ⑴无 ⑵〈1次/周 ⑶1-2次/周 ⑷≥ 3次/周

Had bad dreams (1) Not during the past month (2) Less than once a week (3) Once or twice a week (4) Three or more times a week

1. 疼痛不适 ⑴无 ⑵〈1次/周 ⑶1-2次/周 ⑷≥ 3次/周

Have pain (1)Not during the past month (2) Less than once a week (3) Once or twice a week (4) Three or more times a week

1. 其它影响睡眠的事情 ⑴无 ⑵〈1次/周 ⑶1-2次/周 ⑷≥ 3次/周。如有，请说明。

Other reason(s), please describe____. How often during the past month have you had trouble sleeping because of this? (1) Not during the past month (2) Less than once a week (3) Once or twice a week (4) Three or more times a week

1. 近1个月，总的来说，您认为自己的睡眠质量⑴很好 ⑵较好 ⑶较差 ⑷很差
2. During the past month, how would you rate your sleep quality overall? (1) Very good (2) Fairly good (3) Fairly bad (4) Very bad
3. 近1个月，您用药物催眠的情况⑴无 ⑵〈1次/周 ⑶1-2次/周 ⑷≥ 3次/周
4. During the past month, how often have you taken medicine (prescribed or “over the counter”) to help you sleep?(1) Not during the past month (2) Less than once a week (3) Once or twice a week (4) Three or more times a week
5. 近1个月，您常感到困倦吗 ⑴无 ⑵〈1次/周 ⑶1-2次/周 ⑷≥ 3次/周
6. During the past month, how often have you had trouble staying awake while driving, eating meals, or engaging in social activity? (1) Not during the past month (2) Less than once a week (3) Once or twice a week (4) Three or more times a week
7. 近1个月，您做事情的精力不足吗⑴没有 ⑵偶尔有 ⑶有时有 ⑷经常有
8. During the past month, how much of a problem has it been for you to keep up enough enthusiasm to get things done? (1) No problem at all (2) Only a very slight problem (3) Somewhat of a problem (4) A very big problem

**The Revised Environmental Unpredictability Questionnaire (UP; Luo et al., 2020)**

1. **请思考（回忆你5—14岁时）以下对于你父母的描述是否符合真实情况，“完全不符合”请选择“1”，“比较不符合”请选择“2”，以此类推，“完全符合”请选择“5”。**

Think back to your life when you were 5-14 years. Are the following descriptions of your parents and family true? “strongly disagree” please select “1”, “somewhat disagree” please select “2”, and so on, “strongly agree” please select “5”.

1. 父母在特定情境里的行为取决于他/她的情绪
2. How parents will act in a specific situation depends on their mood.

1 2 3 4 5

1. 父母设定好的规矩常常发生改变
2. Parents keep changing their mind about rules for the children.

1 2 3 4 5

1. 在我调皮时父母是否承接我取决于他们的情绪
2. Whether parents discipline me when I act up depends on their mood at the time.

1 2 3 4 5

1. 我无法预料父母在不同情境里的行为
2. How parents will act from one situation to another is unpredictable.

1 2 3 4 5

1. 有时候父母毫无缘由地对我大喊大叫
2. Sometimes parents yell at me without thinking about what they are saying.

1 2 3 4 5

1. 父母答应我的事情常常无法做到
2. Parents let me get away with breaking the rules.

1 2 3 4 5

1. 我家里的东西总是混乱无序
2. Things were often chaotic in my house.

1 2 3 4 5

1. 父母常常带着我从一个地方搬家到另一个地方
2. My parents often moved with me from one place to another.

1 2 3 4 5

1. 父母常常不在我身边陪伴我
2. My parents are often not around to be with me.

1 2 3 4 5

**The Mini-K Life History Strategy Measure (Figueredo et al., 2014)**

1. **请你指出你对下列陈述认同的程度。使用下列表格并在空白格内填写你的答案。“强烈不同意”请选择“1”，“不同意”请选择“2”，“有点不同意”请选择“3”，“不清楚”请选择“4”，“有点同意”请选择“5”，“同意”请选择“6”，“强烈同意”请选择“7”。**

Please indicate the degree to which you agree or disagree with the following statements. “1=strongly disagree, 2=disagree, 3=slightly disagree, 4=not sure, 5=slightly agree, 6=agree, 7=strongly agree”.

1. 我常常能弄清楚事情将如何发展的
2. I can often tell how things will turn out.

1 2 3 4 5 6 7

1. 为了解决问题，我会设法理解问题出现的原因
2. I try to understand how I got into a situation to figure out how to handle it.

1 2 3 4 5 6 7

1. 我总能看到逆境中积极的一面
2. I often find the bright side to a bad situation.

1 2 3 4 5 6 7

1. 我从不放弃直到问题得到解决
2. I don’t give up until I solve my problems.

1 2 3 4 5 6 7

1. 我时常提前制定计划
2. I often make plans in advance.

1 2 3 4 5 6 7

1. 我避免冒险
2. I avoid taking risks

1 2 3 4 5 6 7

1. 成长过程中，我和亲生母亲关系紧密
2. While growing up, I had a close and warm relationship with my biological mother.

1 2 3 4 5 6 7

1. 成长过程中，我和亲生父亲关系紧密
2. While growing up, I had a close and warm relationship with my biological father.

1 2 3 4 5 6 7

1. 我和我自己的孩子关系亲密
2. I have a close and warm romantic relationship with my sexual partner.

1 2 3 4 5 6 7

1. 我和恋人的关系亲密
2. I have a close and warm romantic relationship with my sexual partner.

1 2 3 4 5 6 7

1. 相比于同时拥有多个性伴侣，我更倾向只有一个
2. I would rather have one than several sexual relationships at a time.

1 2 3 4 5 6 7

1. 只有和对方建立亲密的关系，我才会有舒适的性爱
2. I have to be closely attached to someone before I am comfortable having sex with them.

1 2 3 4 5 6 7

1. 我和自己的亲戚经常交往
2. I am often in social contact with my blood relatives.

1 2 3 4 5 6 7

1. 我的亲戚经常给我情感支持和物质帮助
2. I often get emotional support and practical help from my blood relatives.

1 2 3 4 5 6 7

1. 我经常给我的亲戚情感支持和物质帮助
2. I often give emotional support and practical help to my blood relatives.

1 2 3 4 5 6 7

1. 我和自己的朋友经常交往
2. I am often in social contact with my friends.

1 2 3 4 5 6 7

1. 我的朋友经常给我情感支持和物质帮助
2. I often get emotional support and practical help from my friends.

1 2 3 4 5 6 7

1. 我经常给我的朋友情感支持和物质帮助
2. I often give emotional support and practical help to my friends.

1 2 3 4 5 6 7

1. 我对我生活所在周围环境有归属感
2. I am closely connected to and involved in my community.

1 2 3 4 5 6 7

1. 我忠于自己的信仰
2. I am closely connected to and involved in my religion.

1 2 3 4 5 6 7

**The Perceived Stress Scale (PSS; Cohen et al., 1983)**

1. **请根据过去一个月您的情况，对下面每个阐述，选出最符合您的一项。“从不”请选择“0”，“偶尔”请选择“1”，“有时”请选择“2”，“时常”请选择“3”，“总是”请选择“4”。注意回答这些问题没有对错之分。**

Please think about your feelings and thoughts during the last month. For each statement, please indicate the degree to which you agree or disagree. “0=never, 1=almost never, 2=sometimes, 3=fairly often, 4=very often”. Please note that there are no right or wrong answers to these questions.

1. 一些无法预期的事情发生而感到心烦意乱
2. In the last month, how often have you been upset because of something that happened unexpected?

0 1 2 3 4

1. 感觉无法控制自己生活中的重要事情
2. In the last month, how often have you felt that you were unable to control the important things in your life?

0 1 2 3 4

1. 感到压力和紧张不安
2. In the last month, how often have you felt nervous and "stressed"?

0 1 2 3 4

1. 成功处理恼人的生活麻烦
2. In the last month, how often have you dealt successfully with irritating life hassles?

0 1 2 3 4

1. 感到自己能有效地处理生活中所发生的的重要改变
2. In the last month, how often have you felt that you were effectively coping with important changes that were occurring in your life?

0 1 2 3 4

1. 对于有能力处理自己私人的问题感到很有信心
2. In the last month, how often have you felt confident about your ability to handle your personal problems?

0 1 2 3 4

1. 感到事情顺心如意
2. In the last month, how often have you felt that things were going your way?

0 1 2 3 4

1. 发现自己无法处理所有自己必须做的事情
2. In the last month, how often have you found that you could not cope with all the things that you had to do?

0 1 2 3 4

1. 有办法控制生活中恼人的事情
2. In the last month, how often have you been able to control irritations in your life?

0 1 2 3 4

1. 常觉得自己是驾驭事情的主人
2. In the last month, how often have you felt that you were on top of things?

0 1 2 3 4

1. 常生气，因为很多事情的发生是超出自己所能控制的
2. In the last month, how often have you been angered because of things that happened that were outside of your control?

0 1 2 3 4

1. 经常想到有些事情是自己必须完成的
2. In the last month, how often have you found yourself thinking about things that you have to accomplish?

0 1 2 3 4

1. 常能掌控时间安排方式
2. In the last month, how often have you been able to control the way you spend your time?

0 1 2 3 4

1. 常感到困难的事情堆积如山，而自己无法克服它们
2. In the last month, how often have you felt difficulties were piling up so high that you could not overcome them?

0 1 2 3 4
